# A novel network pharmacology strategy to decode mechanism of Wuling Powder in treating liver cirrhosis

**DOI:** 10.1186/s13020-024-00896-z

**Published:** 2024-03-01

**Authors:** Qinwen Liu, Xiaowei Li, Yi Li, Qian Luo, Qiling Fan, Aiping Lu, Daogang Guan, Jiahui Li

**Affiliations:** 1 Department of Biochemistry and Molecular Biology, School of Basic Medical Sciences, Southern Medical University, Guangzhou, China; 2 Guangdong Provincial Key Laboratory of Single Cell Technology and Application, Southern Medical University, Guangzhou, China; 3 Center for Genetics and Developmental Systems Biology, Department of Obstetrics and Gynecology, Nanfang Hospital, Southern Medical University, Guangzhou, China; 4 Department of Bioinformatics, School of Basic Medical Sciences, Southern Medical University, Guangzhou, China; 5 Institute of Integrated Bioinformedicine and Translational Science, Hong Kong Baptist University, Hong Kong, China; 6 Guangdong-Hong Kong-Macau Joint Lab On Chinese Medicine and Immune Disease Research, Guangzhou, China

**Keywords:** Wuling Powder, Liver cirrhosis, Network pharmacology, Traditional Chinese Medicine

## Abstract

**Background:**

Liver cirrhosis is a chronic liver disease with hepatocyte necrosis and lesion. As one of the TCM formulas Wuling Powder (WLP) is widely used in the treatment of liver cirrhosis. However, it’s key functional components and action mechanism still remain unclear. We attempted to explore the Key Group of Effective Components (KGEC) of WLP in the treatment of Liver cirrhosis through integrative pharmacology combined with experiments.

**Methods:**

The components and potential target genes of WLP were extracted from published databases. A novel node importance calculation model considering both node control force and node bridging force is designed to construct the Function Response Space (FRS) and obtain key effector proteins. The genetic knapsack algorithm was employed to select KGEC. The effectiveness and reliability of KGEC were evaluated at the functional level by using gene ontology (GO) and Kyoto Encyclopedia of Genes and Genomes (KEGG) pathway analysis. Finally, the effectiveness and potential mechanism of KGEC were confirmed by CCK-8, qPCR and Western blot.

**Results:**

940 effective proteins were obtained in FRS. KEGG pathways and GO terms enrichments analysis suggested that effective proteins well reflect liver cirrhosis characteristics at the functional level. 29 components of WLP were defined as KGEC, which covered 100% of the targets of the effective proteins. Additionally, the pathways enriched for the KGEC targets accounted for 83.33% of the shared genes between the targets and the pathogenic genes enrichment pathways. Three components scopoletin, caryophyllene oxide, and hydroxyzinamic acid from KGEC were selected for in vivo verification. The qPCR results demonstrated that all three components significantly reduced the mRNA levels of COL1A1 in TGF-β1-induced liver cirrhosis model. Furthermore, the Western blot assay indicated that these components acted synergistically to target the NF-κB, AMPK/p38, cAMP, and PI3K/AKT pathways, thus inhibiting the progression of liver cirrhosis.

**Conclusion:**

In summary, we have developed a new model that reveals the key components and potential mechanisms of WLP for the treatment of liver cirrhosis. This model provides a reference for the secondary development of WLP and offers a methodological strategy for studying TCM formulas.

**Supplementary Information:**

The online version contains supplementary material available at 10.1186/s13020-024-00896-z.

## Introduction

Liver cirrhosis is a chronic liver disease with hepatocyte necrosis and lesion. Liver ascites will appear when liver cirrhosis develops to the late stage. As of 2019, it was responsible for 2.4% of deaths worldwide [1]. Complex pathogenic mechanism of liver cirrhosis has brought great challenges to clinical treatment. At present, the main treatment methods include using adefovir dipivoxil and entecavir to treat patients with hepatitis B cirrhosis [2], and using spironolactone to treat ascites caused by liver cirrhosis [3]. However, the treatment duration of these antiviral drugs is prolonged, and long-term usage may lead to the development of drug resistance in patients. Additionally, elderly patients with ascites cannot tolerate high-dose spironolactone, and while small-dose spironolactone cannot exert the ideal efficacy. In addition, the efficacy of these treatments is not satisfactory due to the individual variability of patients, and new alternative treatments for cirrhosis are needed.

Traditional Chinese medicine (TCM) formulas increasingly contribute to treating complex diseases, such as liver cirrhosis, by utilizing multi-components, multi-targets and multi-pathways. Song Zhenmin et al. divided 245 patients into six syndrome types according to the diagnosis results, and were treated with Fuzheng Huayu Decoction for 6 months respectively. The changes of liver function indexes and other related indexes before and after treatment were detected. The results showed that the overall effective rates after treatment were 93.2%, 95.3%, 93.6%, 92.9%, 91.7% and 87.9% respectively, and all the indexes improved after treatment [4]. Furthermore, Liu Dezhong et al. demonstrated that after conventional treatment combined with Zhenggan Decoction, the indicators of serum leptin, adiponectin levels and insulin resistance in hepatitis B cirrhosis patients were significantly improved compared with those only using Western medicine [5]. These results reported that TCM formulas are widely used in treating liver cirrhosis, and there are also some prescriptions with good effects.

Among these, Wuling Powder (WLP) has been widely utilized in clinical practice, primarily for its ability to inhibit cell proliferation and inflammatory reactions. WLP is prepared from Cinnamomum cassia Presl (Guizhi, 7 g), Atractylodes macrocephala Koidz. (Baizhu, 10 g), Alisma plantago-aquatica Linn. (Zexie, 15 g), Polyporus umbellatus (Pers.) Fries (Zhuling, 10 g), Poria cocos (Schw.) Wolf. (Fuling, 10 g). Clinical trials have provided evidence of the effectiveness of WLP in the management of cirrhosis. For instance, a study divided 112 patients with ascites caused by hepatitis B cirrhosis into control and treatment groups. The control group received Western medicine, while the treatment group received additional treatment with WLP. The results showed a significantly greater improvement in liver function and liver fibrosis indexes in the treatment group compared to the control group [6]. Additionally, in another study, patients with ascites due to hepatitis B cirrhosis were divided into two groups receiving entecavir dispersible tablets and entecavir dispersion tablets combined with WLP. The results indicated a significant improvement in ALT and AST levels in the WLP group [7]. These clinical trials suggest that WLP has obvious therapeutic effect in the treatment of ascites caused by cirrhosis. However, these reports fail to interpret the key components and mechanism of action of WLP in treating liver cirrhosis from a systematic and overall perspective.

At present, computational pharmacology is an effective tool for us to explore the mechanism of medicinal compounds. It combines network analysis with mathematical modeling method and high-throughput heterogeneous data integration analysis to identify new drug targets and to study the pathogenesis and treatment mechanism of diseases, thus providing new strategies and tools for natural products and TCM formulas to finely adjust the intracellular network of complex diseases, improve drug efficacy and reduce adverse reactions. Liu Wenbin et al. investigated the protective effects of Rabdosia serra on alcoholic liver injury. They speculated and verified the active components and action targets through computational pharmacology [8]. Zhuang Wenbin et al. through the exploration on mechanisms of Wuwei Xiaodu Drink in treating gouty arthritis based on computational pharmacology revealed the action characteristics of Wuwei Xiaodu Drink [9]. However, these are only an interpretation of the mechanism of compound treatment for complex diseases at the qualitative level and do not consider the effects of dose and component concentration in the treatment of the disease.

**Fig. 1:**
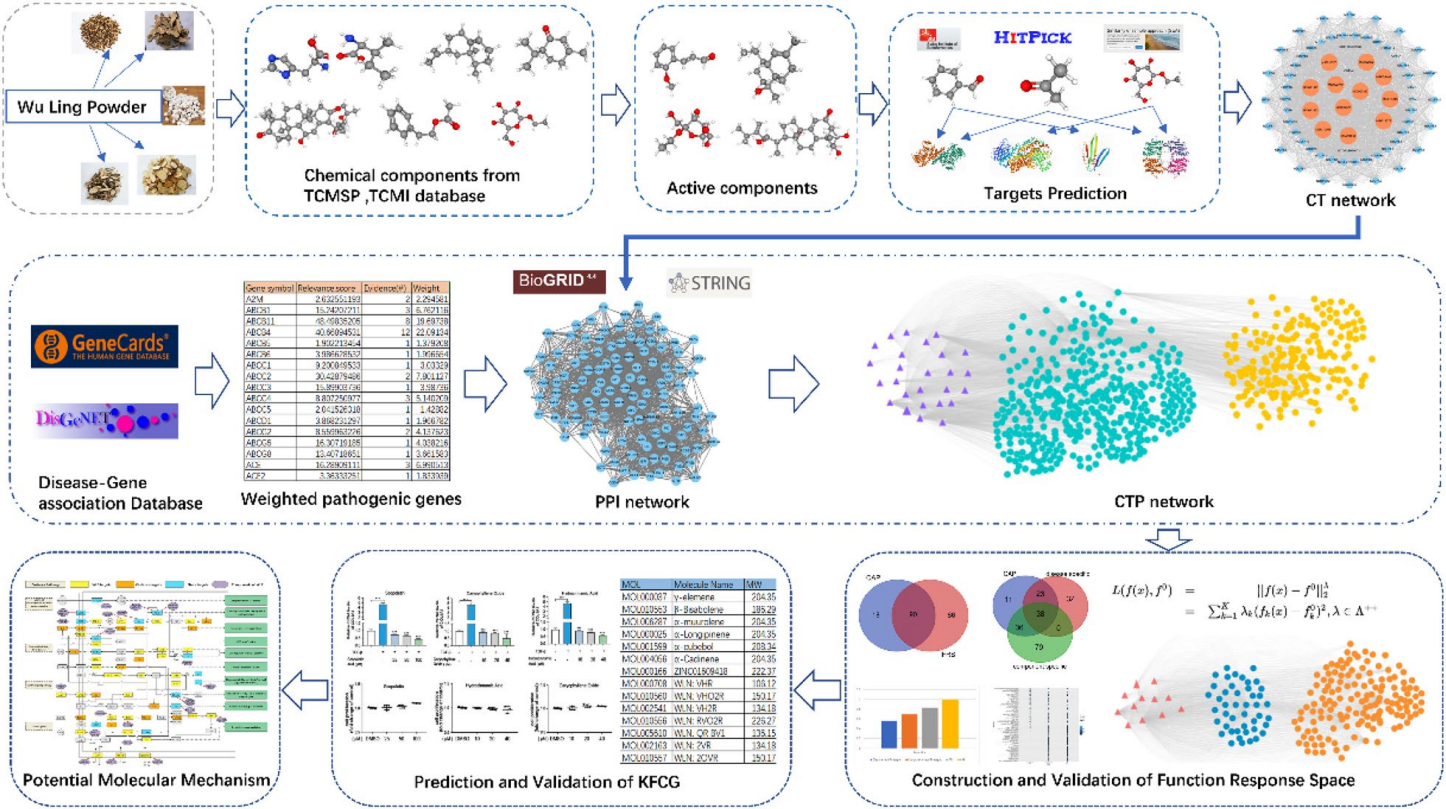
Work scheme for computational pharmacology. Experimental methods include weighted pathogenic genes of liver cirrhosis, C-T network of WLP and compound-target-protein network construction, FRS construction, proteins and mechanism analysis

In order to tackle this issue, we have developed a quantitative computational pharmacology model (Fig. 1). Initially, we collected all the components of WLP from various databases and literature sources. Subsequently, the ADME model was used as a filtering tool to identify the active components among all the compounds. To predict the targets of these active components, we employed SEA, HitPick, and SwissTargetPrediction. By utilizing weighted gene regulatory network and C-T network, we constructed the function response space (FRS) using a multi-objective optimization model, which helped us determine the effective proteins. The KGEC was then obtained through a genetic knapsack algorithm and validated further using KEGG and GO analysis. Lastly, the mechanism of KGEC was confirmed through in vitro experiments. Overall, our strategy provides valuable insights into the analysis of compound optimization mechanisms and pharmacokinetic studies.

## Methods

### The weighted gene regulatory network construction

For the construction of the weighted gene regulatory network for liver cirrhosis, we obtained PPI data in the following databases: BioGRID [10], STRING [11], Dip [12], HPRD [13], Mint [14] and Intact [15]. We extracted and mapped genes linked to liver cirrhosis from GeneCards [16] and DisGeNET [17] onto the PPI network to develop a weighted gene regulatory network tailored to this specific condition. Visualizing the network was achieved using Cytoscape (Version 3.7.1).

### The collection and selection of WLP chemical components

TCM@Taiwan [18], TCMSP [19], TCMID [20], SymMap [21] and ETCM [22] provide all components of the WLP. All components were converted to the SDF format using the Open Babel toolkit (Version 2.4.1), and then their properties were obtained from the SwissADME [23].

### ADME screening

The Lipinski five rules were applied to screen active components of WLP: (1) the compound's molecular weight is below 500 Daltons, (2) donor hydrogen bonds do not exceed 5, (3) there are less than 10 acceptor hydrogen bonds, (4) the lipid-water partition coefficient of the compound is logarithmic and ranges from -2 to 5, (5) it should have 10 or fewer rotatable bonds. Clog P has important effects on drug performance, pharmacokinetics, and toxicology, and serves as a pivotal factor for drug molecule transport from the aqueous phase to the cell membrane [24]. Additionally, we gathered some highly bioactive components from the literature and screened them according to Lipinski five rules. The screened components are used in the follow-up study.

### The prediction for targets of active components

The active components in WLP were analyzed using Similarity Ensemble Approach (SEA) [25], HitPick [26] and SwissTargetPrediction [27] to forecast their targets.

### The definition of the function response space and the evaluation of the effective proteins

FRS was constructed to maximize the search for small molecular targets highly related to pathogenic genes. Initially, we built a network linking active components to their targets. We then analyzed the C-T network and its topological properties using the Cytoscape plugin NetworkAnalyzer. The nodes and their connections in the C-T network were used to create the FRS after conditional screening. The entire process can be outlined as follows:

We defined${Net}_{\text{C-T}}=\{N,E\}$, N means nodes, while ${N}_{\text{com}}$ represents active components, ${N}_{\text{tar}}$ means their targets. E represents the corresponding relationship between active components and targets. ${T}_{tar}=\left\{{P}_{1tar},{P}_{2tar}......{P}_{ntar}\right\}$ is the targets of active component, ${D}_{\text{dis}}=\left\{{G}_{1dis},{G}_{2dis}......{G}_{ndis}\right\}$ is the pathogenic genes of liver cirrhosis. ${C}_{\text{com}}$,${C}_{\text{tar}}$ and ${C}_{\text{dis}}$ respectively represent the number of components, targets and pathogenic genes. ${C}_{com{\prime}}$ and ${C}_{tar{\prime}}$ respectively represent the number of selected components and selected targets. ${C}_{{T}_{tar}\cap {D}_{dis}}$ means the number of genes shared by targets set and pathogenic genes set. We used deviation variables to represent the difference between the actual value and the objective value. d^+^ is positive deviation variable and d^−^is passive deviation variable. Three objective functions were defined, multiple component sets were selected through the following formulas, and finally the component subset with the maximal degree was extracted to obtain the function response space.

$${{\text{min}}Z ={p}_{1}{d}_{1}^{-}+p }_{2}{d}_{2}^{-}+{p }_{3}{d}_{3}^{+}$$

$$s.t.\left\{\begin{array}{c}\begin{array}{c}{C}_{com{\prime}}+ {C}_{tar{\prime}}+ {d}_{1}^{-}- {d}_{1}^{+}= {C}_{\text{tar}}*70\%\\ {C}_{com{\prime}}+ {d}_{2}^{-}- {d}_{2}^{+} = {C}_{{T}_{tar}\cap {D}_{dis}}*80\%\\ {C}_{com{\prime}}{+ d}_{3}^{-}- {d}_{3}^{+} \le {C}_{\text{com}}*40\%\end{array}\\ {C}_{com{\prime}} , {C}_{tar{\prime}} \ge 0\\ {d}_{1 }, {d}_{2},{d}_{3}\ge 0\end{array}\right.$$

$$FRS={Net}_{{d\left[\frac{{C}_{com{\prime}}!}{{C}_{com{\prime}}!\left({N}_{com{\prime}}-{C}_{com{\prime}}\right)!}\right]}_{max}}$$

In which ${d\left[\frac{{C}_{com}{\prime}!}{{C}_{com{\prime}}!\left({N}_{com{\prime}}-{C}_{com{\prime}}\right)!}\right]}_{max}$ is the maximum degree of component subsets. FRS represents the C-T network with the maximum degree, and the C-T network was identified as the function response space.

### Determine a key group of effective components based on a function response space

The genetic knapsack algorithm was used to obtain KGEC in FRS to reveal the mechanism of WLP for the treatment of liver cirrhosis. The calculation process is as follows:

1. Binary coding: transforming the problem space into genetic space. Chromosome coding method for knapsack problem: each object to be solved is represented as a binary string of length n. ${x}_{i}=0$, indicating that the binary code is 0, and vice versa.
2. Generating initial population: randomly generating a population consisting of multiple chromosomes.
3. Calculation of population fitness: on the premise that the value does not exceed C, ${v}_{i}$ and ${w}_{i}$ are used to calculate the fitness of individuals in the population.
4. Selection: the individual fitness is proportionately converted into the roulette area using the roulette model. The chromosome corresponding to the region where random number is located is then selected.
5. Crossover: randomizing crossover points for selected chromosomes and swapping some genes between the paired chromosomes.
6. Mutation: generate random numbers to determine the gene mutation location on each chromosome and invert the original gene value at the mutation site.
7. Iteration: after forming a new population, return to the third step to continue iteration until the termination condition of $W=C$ is met.
  $$maxV={\sum }_{i=1}^{n}{v}_{i}{x}_{i}$$
  $$W=\sum_{i=1}^{n}{w}_{i}{x}_{i}\le C$$
  $${x}_{i}= 0/1,i=\mathrm{1,2},3,\dots ,n$$

### Experimental verification

#### Cell culture and reagents

LX-2 cells were grown in Dulbecco’s modified Eagle’s medium (Invitrogen, Shanghai, China), with the addition of 10% fetal bovine serum (FSP500, ExCell bio, ShangHai), 100 μg/mL streptomycin, and 100 μg/mL penicillin in a humidified environment with 5% CO2 at 37 °C. scopoletin, caryophyllene oxide, and hydrocinnamic acid (≥ 99% purity by HPLC) were bought from TargetMol (USA) and dissolved in dimethyl sulfoxide (Sigma, USA). TGF-β1 was acquired from PeproTech, Inc. (Suzhou, Jiangsu, China).

#### Cell viability assay

In brief, LX-2 cells were seeded in 96-well plates. After starving the cells with serum-free DMEM for 6 h, 10 ng·mL^−1^ TGF-β1 was added. 24 h later, cells were treated with scopoletin, caryophyllene oxide and hydrocinnamic acid for 48 h. Subsequently, 10 μL CCK-8 reagent (New Cell & Molecular Biotech) was added to each well, and the cells were further incubated for 2 h in a constant-temperature incubator. The absorbance of the solution at 450 nm was then measured to quantify the results. The experiments were carried out in triplicate.

#### Quantitative real-time PCR (qPCR)

Total RNA was extracted using the Total RNA Isolation Reagent Kit (Magen, China) according to the manufacturer’s protocol. RNA was reverse-transcribed into cDNA using the TransScript® Uni All-in-One First-Strand cDNA Synthesis SuperMix for qPCR kit (TransGen Biotech, China) and quantitative PCR was performed using SYBR Premix (Vazyme Biotech Co., Ltd.). The specific primer sequences for each mRNA were listed in Additional file 1: Table S1. All the experiments were performed on QuantStudio 1.

### Gene ontology and pathway analysis

Gene Ontology (GO) and KEGG pathway analysis was performed for targets by R package clusterProfiler. *P* value was set to 0.05 as the cut-off criterion. The results of KEGG pathway analysis were annotated by R package Pathview [28].

### Western blot

Total protein was extracted using RIPA lysis buffer. Equal amounts of protein samples (20–30 μg) were separated by SDS-PAGE and transferred onto a polyvinylidene fluoride (PVDF) membrane. After blocking with 5% skim milk for 1 h, the membrane was incubated with the primary antibody overnight at 4 °C. Then, the membrane was washed three times with TBST for 5 min each time, followed by incubation with the secondary antibody at room temperature for 1 h. The antibody signals were detected using an electrochemiluminescence substrate (Abbkine Scientific Co., Ltd, China). The antibody list is provided below: β-actin (cat. no. #13E5), NF-κB (cat. no. 8242 T), phospho-NF-κB (cat. no. 3033 T), p38 (cat. no. 8690 T), phospho-p38 (cat. no. 4511 T), AKT (cat. no. 4691 T), phospho-AKT (cat. no. 4060 T), PKA C-α (cat. no. 5842 T), phospho-PKA C (cat. no. 5661 T). The uncropped original image is shown in Additional file 4: Figure S1.

## Results

### Construction of the weighted gene regulatory network for liver cirrhosis

**Fig. 2:**
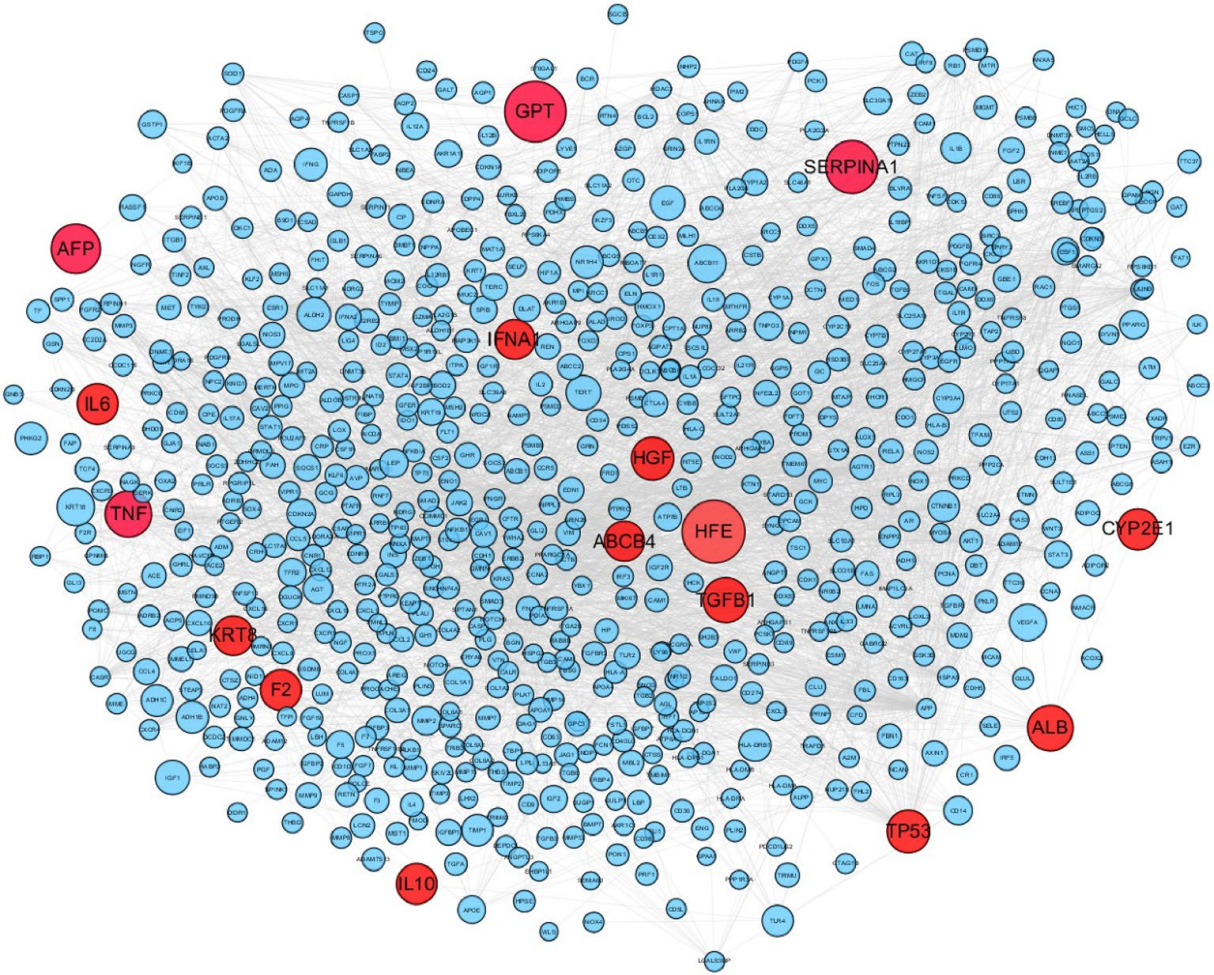
Weighted gene regulatory network of cirrhosis. Node size represents the relevance score of pathogenetic genes multiplied the number of literatures of liver cirrhosis, followed by extracting the square root. The red nodes indicate the top 16 pathogenetic genes with the highest weight in this network

A key step in understanding the pathogenesis of liver cancer and developing intervention strategies is the construction and analysis of weighted gene regulatory networks. The PPI network was made up of public PPI databases, including BioGRID, STRING, Dip, HPRD, Mint and Intact. To construct the weighted gene regulatory network for liver cirrhosis, 897 pathogenic genes associated with liver cirrhosis were obtained from GeneCards and DisGeNET (Additional file 2: Table S2) and mapped to the PPI network. The weighted gene regulatory network consisted of 744 nodes and 4201 edges (Fig. 2). Among the top 30 genes in the gene regulatory network, 7 genes are enriched in alcoholic liver disease (hsa04936), including TNF, IL6, IFNA1, CYP2E1, ALDH2, ADH1B, ADH1C. Alcoholic liver disease usually develops fatty liver initially and can develop into alcoholic hepatitis, hepatic fibrosis and cirrhosis. The result indicated that the weighted gene regulatory network together with the weighted gene are capable of reflecting the underlying pathogenesis of liver cirrhosis. This information serves as a dependable reference for the subsequent construction of the functional response space.

### Collection of chemical components and active components screening

Chinese medicinal formulation contains multiple components, but not all of components are satisfied with pharmacokinetic screening characteristics. According to the Lipinski five rules, 267 active components were selected out from 386 components of WLP (Additional file 3: Table S3). Among 267 active components, GZ includes 194 active components, BZ includes 43 active components, ZX includes 23 active components, FL includes 7 active components and ZL includes 9 active components (Table 1). The chemical composition analysis provides a reference for selecting the active components of WLP to further analyze. The components with high concentration in the prescription usually have certain chemical activity, so we collected the chemical components with high concentration verified by experiments from the literature. The concentration of 25 components in the chromatograms of WLP were found (Table 2).

**Table 1:** WLP components collected in the public database and active components after selecting

Herbs	Components	Active components
Cinnamomum cassia Presl (GZ)	220	194
Atractylodes macrocephala Koidz. (BZ)	55	43
Alisma plantago-aquatica Linn. (ZX)	46	23
Poria Cocos (Schw.) Wolf. (FL)	34	7
Polyporus umbellatus (Pers.) Fries (ZL)	31	9
Total	386	276

**Table 2:** Collection and concentration arrangement of chemical components

Formula	Method	Component	Concentration (mg/g)	Refs
Wuling Powder	HPLC	Alisol A 24-acetate	0.022 ± 0.000816	[29]
		Alisol B 23-acetate	0.061 ± 0.001247	
Cinnamomum cassia Presl	HPLC	Coumarin	0.26	[30]
		Cinnamic Acid	0.49	
		Cinnamaldehyde	6.72	
		Cinnamic Alcohol	0.17	
Atractylodes macrocephala Koidz	HPLC	Atractylenolide I	0.375	[31]
		Atractylenolide II	0.334	
		Atractylenolide III	0.578	
		Atractylone	1.36	
Alisma plantago-aquatica Linn	HPLC	Alisol A	0.193	[32]
		Alisol F	1.69	
		Alisol A 24-alcohol	0.532	
		Alisol B 23-alcohol	0.448	
Poria cocos (Schw.) Wolf	HPLC	Dehydrotumulosic Acid	0.3434	[33]
		Dehydropachymic Acid	0.296	
		Pachymic Acid	0.7282	
		Pinic Acid	0.1499	
Polyporus umbellatus (Pers.) Fries	HPLC	Polyporone A	4.31 × 10^–4^	[34]
		Polyporone B	9.2 × 10–4	
		Polyporone C	9.37 × 10–4	
		Polyporone D	1.083 × 10–3	
		Steroid	3.003 × 10–3	
		ergosterol-7,22-dien-3-ketone	3.46 × 10–4	

### Shared components of herbs in WLP

**Fig. 3:**
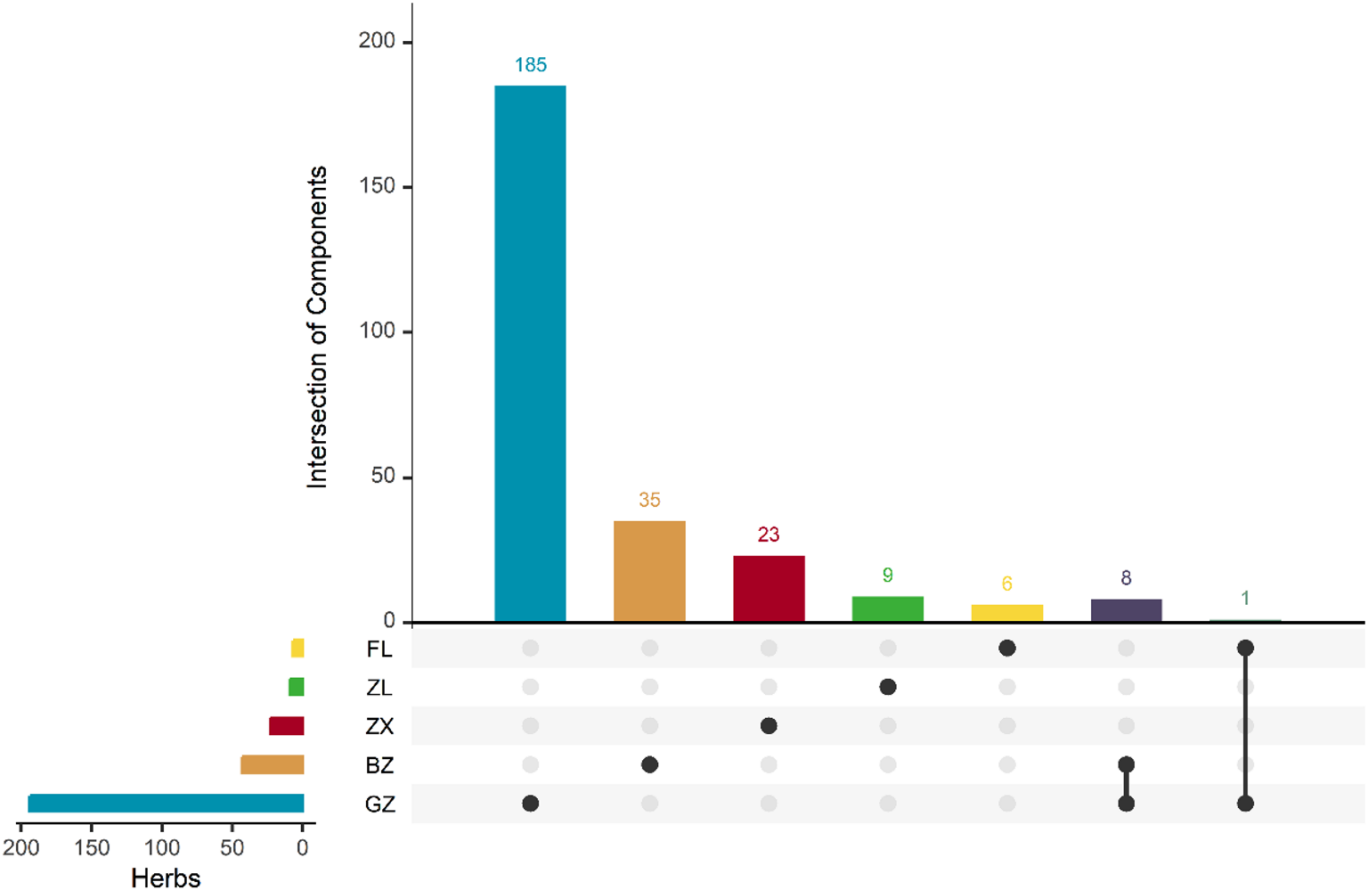
The UpSet picture of WLP components

There are 9 shared components of two herbal medicines in WLP (Fig. 3). For example, caprylic acid (WLP1) is a common component in two herbal medicines (GZ, FL). Caprylic acid is a medium-chain fatty acid (MCFAs). The in vitro experiments with purified BCKDC-BDK complex showed that caprylic acid inhibited BDK activity. Rats were orally administrated with caprylic acid to down-regulate BDK activity by reducing the number of BDK binding complexes, thereby activating liver BC-KDC, decreasing plasma BCAA concentration and increasing serum ketone body concentration [35]. Hemo-sol (D-limonene, WLP7) is a common component of two herbal medicines (GZ and BZ). It has been found to be effective in preventing carbon tetrachloride-induced liver fibrosis in rats by controlling oxidative stress and suppressing the inflammatory pathway. Furthermore, it can regulate the expression of markers associated with fibrosis, such as TGF-b and hydroxyproline [36].

### Specific components of herbs in WLP

Most herbs have their own specific components in addition to the common ones. L-lysin (WLP207), a specific component of BZ, is able to regulate the increase in B-oxidation cells in the liver of SAMP8 mice to which the L-lysin-rich feed was added, suggesting that the dietary intake of lysine could prevent liver steatosis by stimulating B-oxidation in SAMP8 mice [37]. Specific component NON (Decanoic acid, WLP116) of GZ could be converted into decanoate, which upregulates the expression of adipogenic transcription factors and enzymes to reduce fat formation [38]. HCI (3-phenylpropionic acid, WLP163), which is a particular component of GZ, can reduce the CISP-induced oxidative stress in liver. This is achieved by limiting inflammatory signals like COX-2 and NF-κB, as well as apoptotic signals including BAX and caspase-3. Moreover, it enhances the expression of Bcl-2. [39].

### C-T Network construction of active components

The treatment of complex diseases with TCM formulas involves the chemical components and their targets. A single component can correspond to multiple targets, and likewise, a single target can be targeted by multiple components. Therefore, it can be viewed as a network of multiple components and multiple targets, known as the C-T network. To study the mechanics of WLP in addressing liver cirrhosis, the C-T network was established using 267 active components along with 1372 targets. Due to the potential for multiple targets corresponding to a single component, there is a total of 10,849 relationship pairs existing between the mentioned components and their respective targets. The mean number of targets per component was 40.63, indicating the multi-target properties of WLP in treating liver cirrhosis. Caprylic acid (WLP1, degree = 252) has the most targets, with Benzyl acetate (WLP65, degree = 248), WLN: 2OVR (formic acid, WLP30, degree = 244), HCI (3-phenylpropionic acid, WLP163, degree = 252), BZM (benzoic acid, WLP83, degree = 220), and PHA (phenylalanine, WLP218, degree = 214) following closely behind.

The average target degree of the components is 7.91 in the C-T network. The top 20 by weight are MAPT, CA3, CES1, CES2 and MOAB, etc. These targets have been reported to be related to liver diseases and may play a crucial role in the therapeutic effects of WLP in treating liver cirrhosis. Research has indicated that MAPT is able to predict the prognosis of patients with liver cancer [40]. CA3 can inhibit the proliferation of hepatoma HepG2 cells and induce their apoptosis [41]. For immune liver injury, the expression and metabolic activity of CES1 and CES2 are significantly decreased [42]. MAOB is involved in the biosynthesis of endogenous geranyl valeric acid (GGA) through geranyl geraniol oxidation, while GGA is a prophylactic agent for secondary primary liver cancer [43]. Overall, these results indicate that WLP can treat liver cirrhosis via multiple targets and confirm its multi-targeted role in treatment.

### Determination and verification of function response space

We established a multi-objective optimization model including three customized objective functions, to identify the subset with the largest degree from the C-T network as the FRS. The FRS consists of 993 nodes and 2493 edges. 940 nodes from the targets and 53 nodes from the components, thus we defined 940 effective proteins from the FRS.

**Fig. 4:**
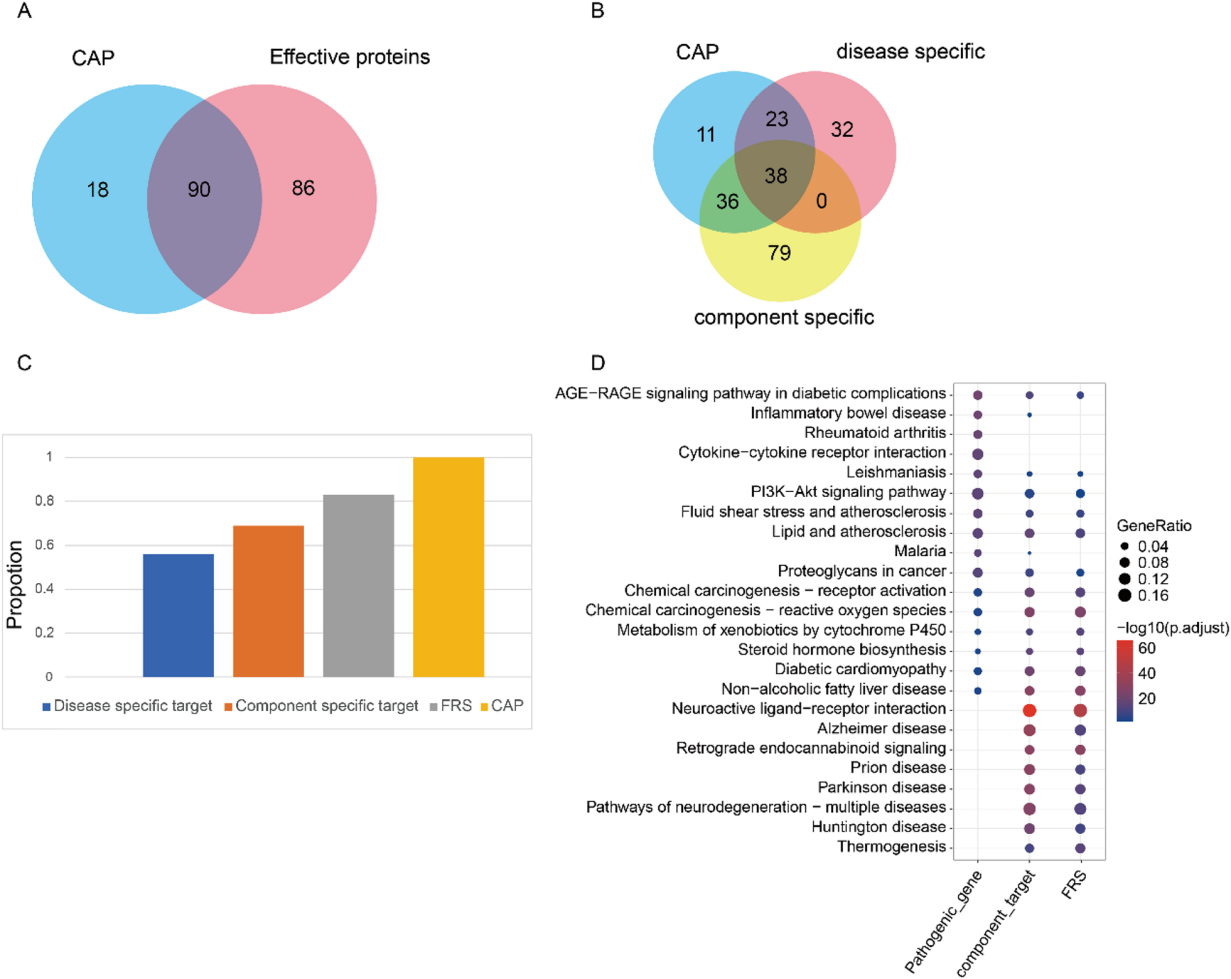
Verification of Function Response Space. **A** Venn diagram shows the number of pathways enriched by effective proteins and the shared genes of CAP. **B** Venn diagram shows the number of pathways enriched by component-specific targets, disease-specific targets and CAP. **C** Proportion histograms depict the enriched pathways of component-specific targets, disease-specific targets and effective proteins in FRS and CAP. **D** Bubble diagram shows the shard pathway enriched by effective proteins, component targets and disease targets (liver cirrhosis pathogenic genes)

To determine whether the effective proteins identified from the FRS adequately representing the pathogenic genes of cirrhosis at a functional level, we conducted functional pathway analyses using effective proteins, components, and pathogenic genes specific to cirrhosis. Notably, the shared genes of targets and pathogenic genes (CAP) were enriched in 108 KEGG pathways (*P* < 0.05), while effective proteins were enriched in 176 KEGG pathways (*P* < 0.05), with effective proteins’ enrichment pathways expected to encompass 83% of the pathways enriched by CAP (Fig. 4A). Additionally, KEGG pathway analyses were performed on disease-specific targets and component-specific targets to assess whether the FRS could be substituted by these targets. Disease-specific targets covered 56% of the pathways enriched by CAP, and component-specific targets covered 69% of these pathways (Figs. 4B and C), which were markedly lower than the coverage of effective proteins. Furthermore, effective proteins’ pathways were largely included in both component target pathways and disease target pathways (Fig. 4D). These findings validate the accuracy and robustness of our multi-objective optimization and establish that the effective proteins identified in the FRS play a crucial role in the pathogenesis of cirrhosis.

According to KEGG pathway analysis, these potent proteins were involved in non-alcoholic fatty liver disease (hsa04932), metabolism of xenobiotics by cytochrome P450 (hsa00980), drug metabolism-cytochrome P450 (hsa00982) and VEGF signaling pathway (hsa04370) (Fig. 4D). For example, non-alcoholic fatty liver disease (hsa04932) suggests that non-alcoholic fatty liver disease may further contribute to liver cirrhosis. After the formation of non-alcoholic fatty liver disease, ROS production is enhanced due to mitochondrial β-oxidation of fatty acids and oxidative stress of endoplasmic reticulum stress, leading to lipid peroxidation. Lipid peroxidation further leads to the production of cytokines (Fas ligand, TNF-α, IL-8, and TGF) and promotes cells death, inflammation, and fibrosis [44]. Polymeric immunoglobulin receptor (PIGR) is significantly increase in non-alcoholic fatty liver disease and cirrhosis. The strategy of using the multi-objective optimization model phase to optimize the herbal formulation is reliable, and the FRS may play a part in by mediating multiple metabolic and inflammation-related pathways.

### KGEC selection and validation

The knapsack algorithm model was applied to optimize the active components group and obtain the KGEC. A total of 29 components were chosen as the KGEC, as they collectively contributed to 100% coverage of the targets of effective proteins (Table 3).

**Table 3:** The information of KGEC in WLP

ID	Component	MW	ID	Component	MW
WLP161	(-)-Epoxycaryophyllene	220.35	WLP217	LPG	89.09
WLP9	( ±)-Isoborneol	154.25	WLP171	Methylcinnamate	162.19
WLP226	(1R)-2-methyl-1-phenylprop-2-en-1-ol	148.2	WLP230	NCA	122.12
WLP152	(1R,4R)-4-isopropyl-1,6-dimethyltetralin	202.34	WLP218	PHA	165.19
WLP172	(Z,Z)-farnesol	222.37	WLP194	PHB	138.12
WLP249	1 h-indole-3-carboxylic,acid	161.16	WLP264	polyporusterone,c	476.65
WLP255	2-lauroleic acid	198.3	WLP193	protocatechuic acid	154.12
WLP221	Akridin	179.22	WLP179	Safrol	162.19
WLP232	alisol B	444.65	WLP219	Scopoletol	192.17
WLP228	alpha-humulene	204.35	WLP254	Undekansaeure	186.29
WLP175	BOX	122.12	WLP204	uridine	244.2
WLP2	DIBP	278.34	WLP209	GLY	75.07
WLP206	DTY	181.19	WLP208	Glutamine	147.13
WLP253	Ethyl glucoside	208.21	WLP163	HCI	150.17
WLP168	eugenol	164.2			

**Fig. 5:**
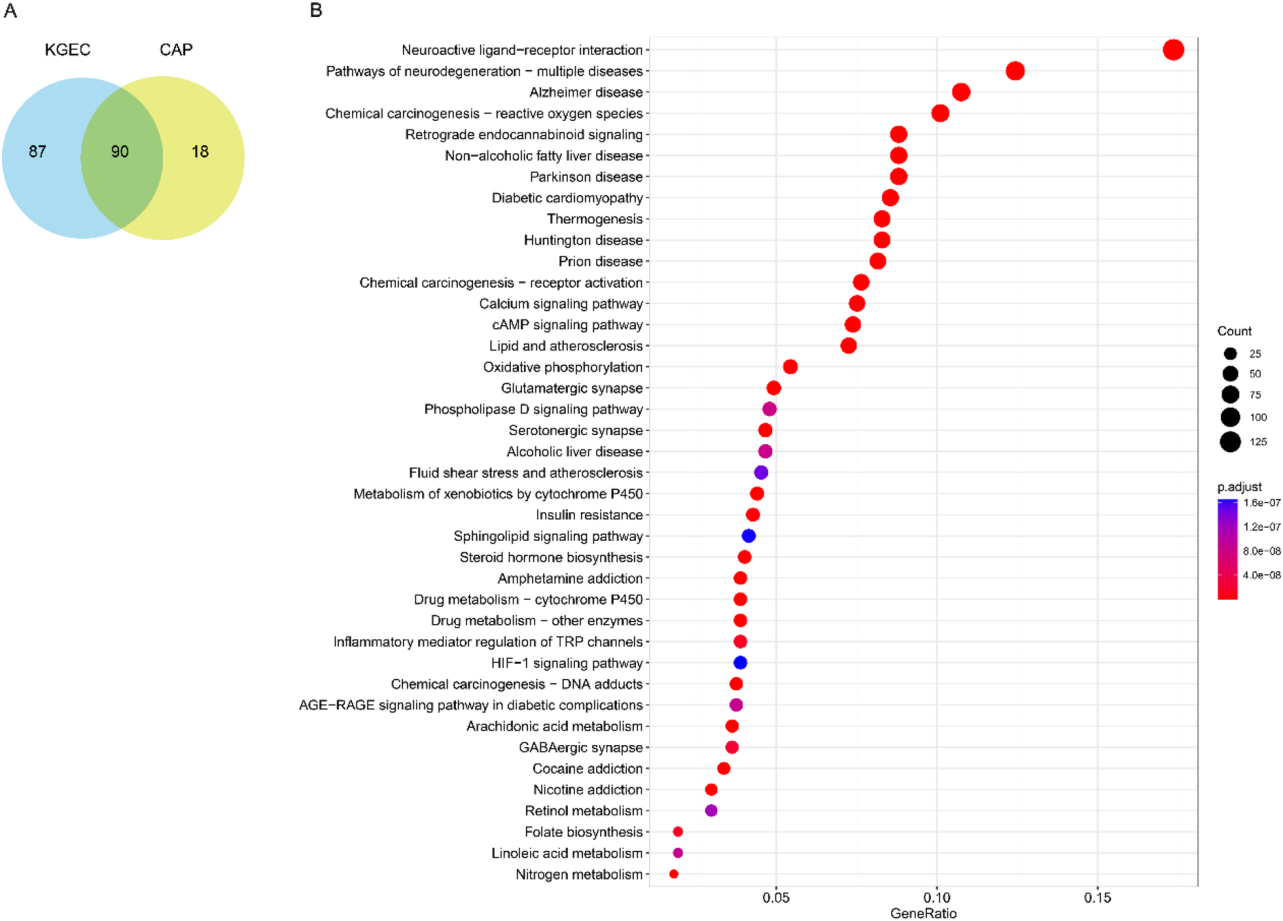
Pathway enrichment analysis of KGEC targets. **A** Venn diagram for KGEC targets and CAP targets through pathway enrichment analysis. **B** The size of the circle expresses gene’s number enriched in the pathway, and the color expresses the significance of genes enriched in the pathway

We conducted KEGG pathway analysis for KGEC targets and CAP targets separately to examine the potential role of WLP in treating liver cirrhosis. 177 pathways were found to be enriched in KGEC targets (P < 0.05), while 108 pathways were enriched in CAP targets (P < 0.05). Notably, the pathways enriched in KGEC targets covered 83.33% of those enriched in CAP targets (Fig. 5A). The majority of the KEGG targets were involved in pathways such as non-alcoholic fatty liver disease (hsa04932), thermogenesis (hsa04714), cAMP signaling pathway (hsa04024), chemical carcinogenesis-reactive oxygen species (hsa05208), cytochrome P450 metabolism of xenobiotics (hsa00980), drug metabolism-cytochrome P450 (hsa00982), and oxidative phosphorylation (hsa00190) (Fig. 5B). Of these, the non-alcoholic fatty liver pathway (hsa04932) results in an increase in intracellular fatty acid-derived metabolites due to a lack of insulin receptor substrate-2 (IRS-2)-associated phosphatidylinositol 3-kinase (PI3K) activity. This damages the mitochondria of NASH patients, which in turn promotes liver fibrosis. [44]. These findings suggest that combining the FRS with the knapsack algorithm model for optimizing the herbal formulation is reliable, and the predicted KGEC may have a therapeutic effect by participating in several signal transduction and apoptosis pathways.

### Cellular experimental verification of KGEC

**Fig. 6:**
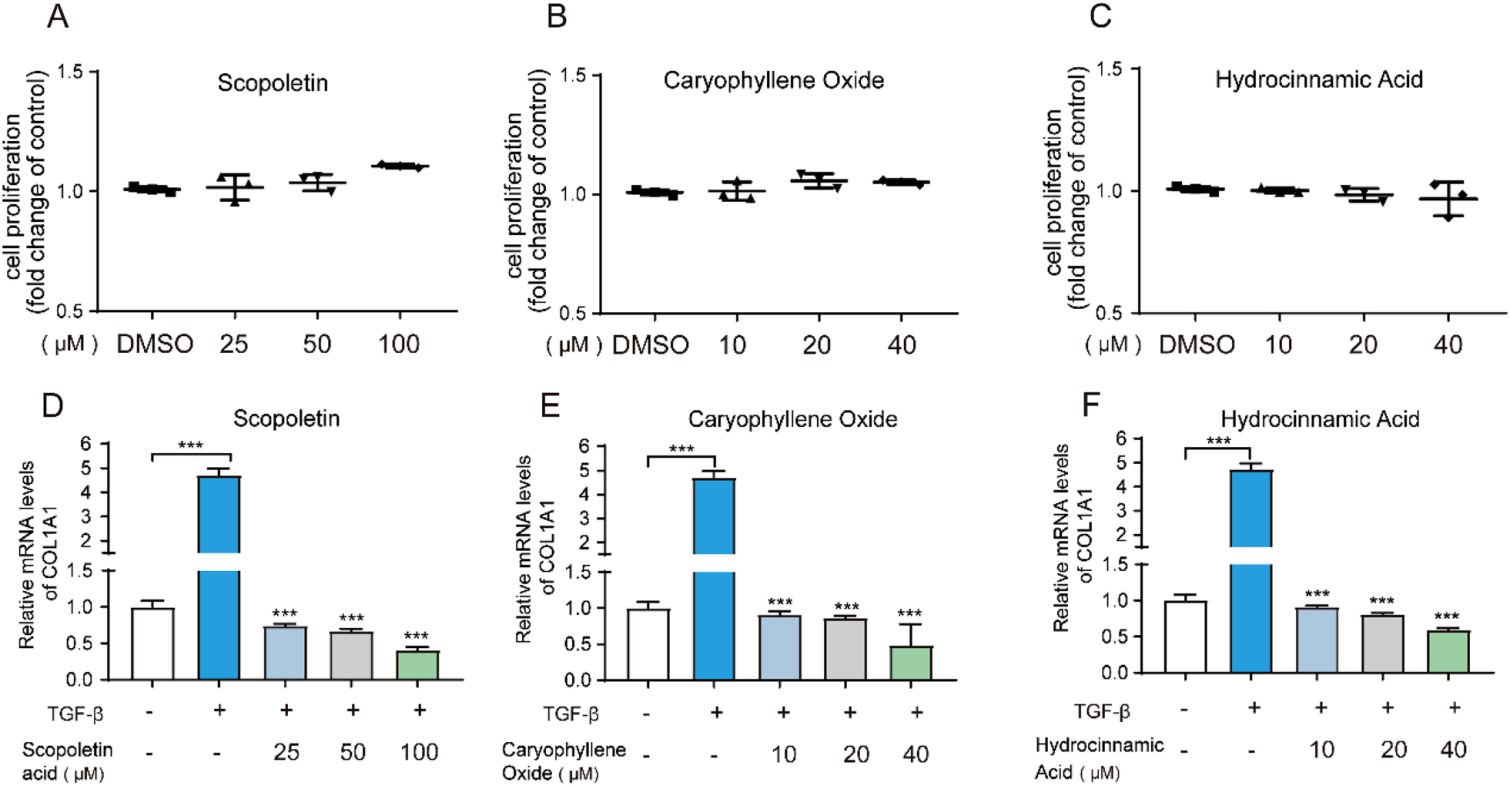
**A-C** Cellular experimental verification of KGEC. LX-2 cells were treated with scopoletin, caryophyllene oxide and hydroxyzinamic acid for 48 h and the cell viability was detected by CCK-8 with DMSO as control. **D-F** The mRNA levels of COL1A1 were detected by qPCR after LX2 cells were serum-starved for 6 h, induced with TGF-β1 for 24 h, and then treated with the three components at indicated concentrations for 48 h

Simple random sampling is a form of probability sampling. The probability that each sample drawn is equal, and each unit of the sample is completely independent, thus can exclude the correlation and exclusion between samples [45]. Based on this strategy, we selected scopoletin, caryophyllene oxide and hydroxyzinamic acid in KEGC for in vitro experimental validation. Here, we used TGF-β1-induced LX2 cells as a model of liver fibrosis, a widely recognized and employed model. First, through CCK8 experiments, we determined that scopolamine was safe at concentrations below 100uM, while saponin and hydroxycinnamic acid were safe at concentrations below 40uM (Fig. 6A-C). Subsequently, we successfully induced the LX2 cell model of liver fibrosis, as evidenced by a significant increase in the expression of the liver fibrosis marker gene COL1A1 (Fig. 6D-F). We found that, at safe concentrations, all three components significantly suppressed the expression of COL1A1 in a concentration-dependent manner (Fig. 6D-F). These results indicate that scopolamine, saponin, and hydroxycinnamic acid can significantly inhibit TGF-β1-induced liver fibrosis in LX2 cells, without inducing significant cytotoxicity. This further demonstrates the therapeutic effect of the KEGC selected from the FRS in treating liver fibrosis.

### GO enrichment analysis of KGEC targets

**Fig. 7:**
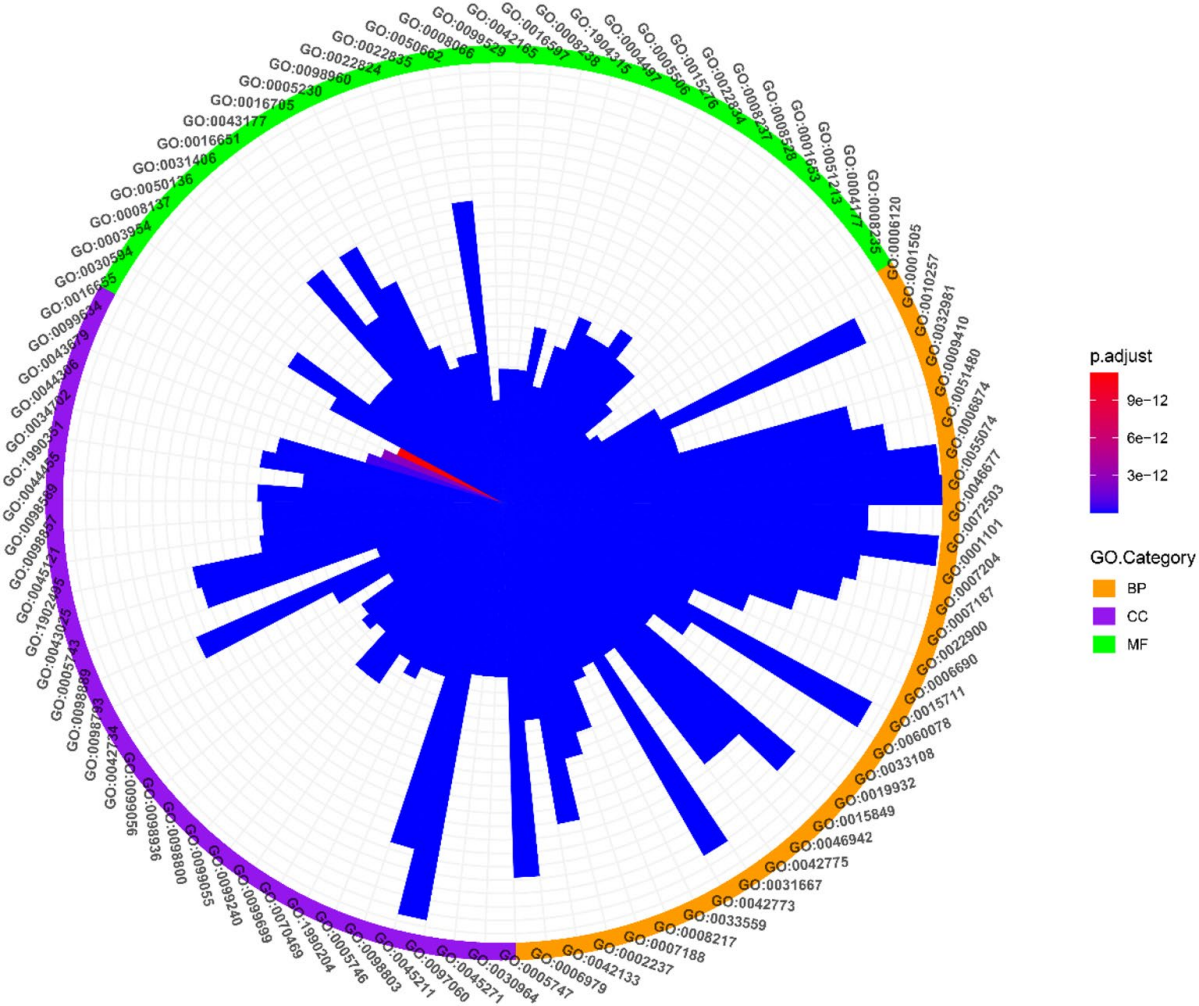
GO enrichment analysis of KGEC targets. The colors orange, purple, and green represent biological processes, cellular components, and molecular functions, respectively. The varying colors in the bar graph indicate the significance of gene enrichment in GO

To further investigate the combined effects of WLP, we conducted GO enrichment analysis on all the targets interacting with KGEC (Fig. 7). The findings revealed that KGEC targets were enriched in biological processes associated with mitochondrial electron transfer, including mitochondrial electron transfer, NADH to ubiquinone (GO:0006120, involving SNCA, NDUFB5, NDUFB7, NDUFB3, etc.), and mitochondrial respiratory chain complex assembly (GO:0033108, involving UQCRB, NDUFB5, NDUFB7, etc.), as well as mitochondrial ATP synthesis coupled electron transport (GO:0042775, including UQCRB, SNCA, NDUFB5, NDUFB7, etc.). Additionally, KGEC targets were enriched in cellular components related to the respiratory chain, such as mitochondrial respiratory chain complex I (GO:0005747, NDUFB5, NDUFB7, NDUFB3, NDUFB4, etc.), respiratory chain complex (GO:0098803, UQCRB, NDUFB5, NDUFB7, NDUFB3, etc.), and NADH dehydrogenase complex (GO:0030964, etc., NDUFA10, NDUFB2, etc.), while also participating in the regulation of redox-related enzyme activities, such as NADH dehydrogenase activity (GO:0003954, NDUFB5, NDUFB7, NDUFB3, NDUFB4, etc.), and oxidoreductase activity, acting on NAD(P)H, quinone or similar compound as acceptor (GO:0016655, NQO2, CBR1, NQO1, AKR1C4, etc.). It is noteworthy that accumulating evidence suggests a close association between mitochondrial dysfunction and the pathogenesis of liver cirrhosis, with oxidative stress promoting liver inflammation and fibrosis, and poorly regulated mitochondria leading to the overproduction of reactive oxygen species [46].

Additionally, the targets of KGEC were enriched for pathways that were associated with inflammation, including G protein-coupled receptor signaling pathway coupled to cyclic nucleotide second messenger (GO:0007187, involving OPRK1, ADRA2C, ADRB1, CCR, etc.), second messenger-mediated signal transduction (GO:0019932, with ADRA2C, ADRB1, MAPT, CCR3, etc.), adenylate cyclase-modulating G protein-coupled receptor signaling pathway (GO:0007188, including OPRK1, ADRA2C, ADRB1, CCR3, etc.), and phospholipase C-activating G protein-coupled receptor signaling pathway (GO:0007200, with ESR1, OPRK1, CX3CR1, C3AR1, etc.). It is important to note that the transition from chronic liver disease to liver cirrhosis involves inflammation, activation of liver cirrhosis cells, and simultaneous cortical extinction due to fibrosis angiogenesis, and vascular occlusion [47]. Studies have demonstrated that mitochondrial-DAMP released by damaged hepatocyte mitochondria triggers a potent inflammatory reaction, leading to the direct activation of liver cirrhosis cells and liver fibroblasts, resulting in the formation of liver scars and ultimately leading to cirrhosis [48]. Therefore, inflammation plays a crucial role in the development of cirrhosis. Overall, the GO analysis suggests that WLP may alleviate liver cirrhosis by modulating the biological process of electron transport in mitochondria and inflammation.

### KEGG enrichment analysis of KGEC targets

**Fig. 8:**
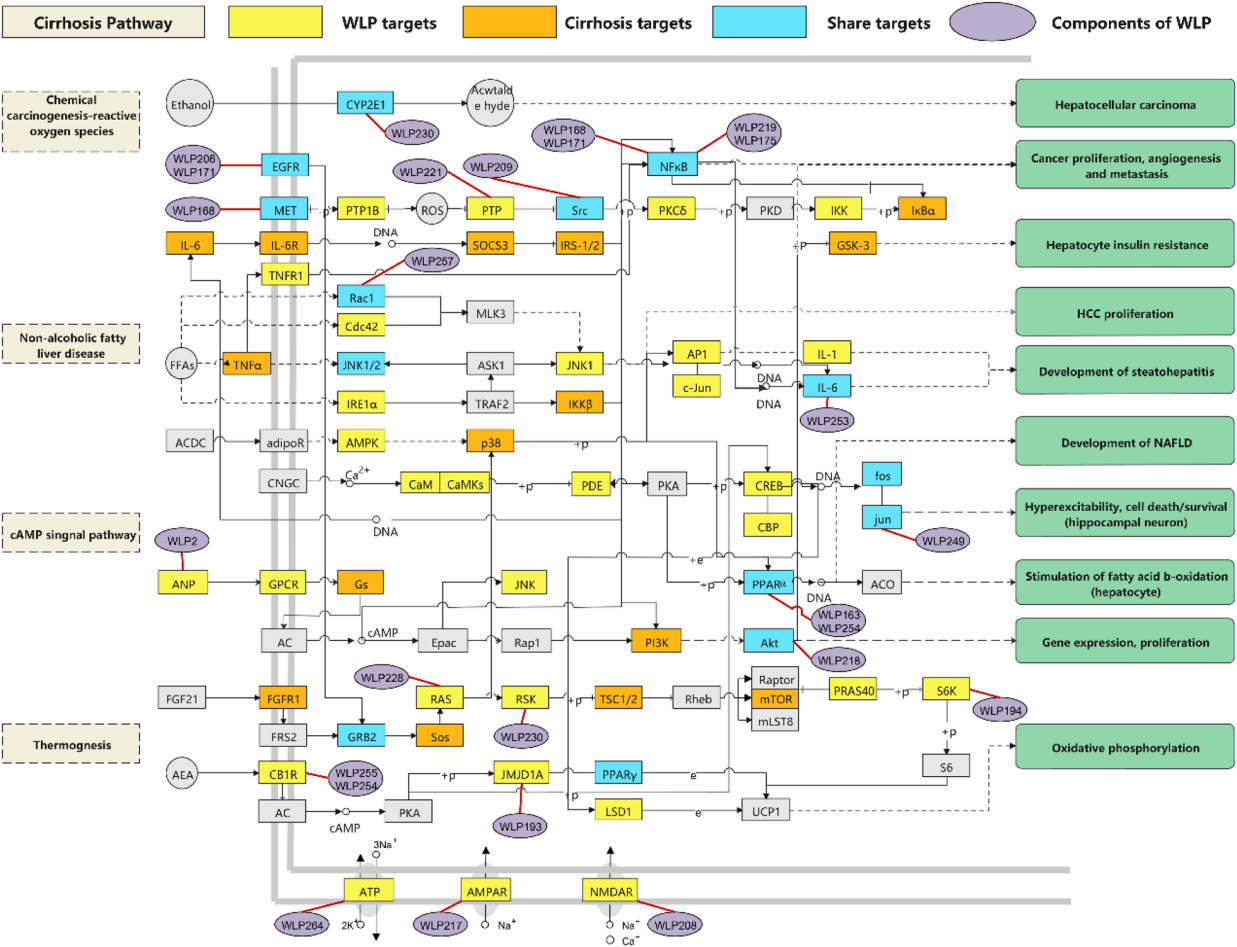
Distribution of KGEC targets on integrated signaling pathways. Various colors represent different target types. Yellow represents WLP targets, orange depicts the pathogenic genes of cirrhosis, blue refers to the targets shared by WLP and the pathogenic gene of cirrhosis and purple represents the components of WLP

Liver cirrhosis is now recognized as a dynamic process rather than a terminal disease. A clinical sub-classification of cirrhosis prognosis has been proposed, delineating four distinct stages with varying mortality rates [54]. Growing evidence suggests that non-alcoholic fatty liver disease pathway (hsa04932), thermogenesis (hsa04714), cAMP signaling pathway (hsa04024), and chemical carcinogenesis-reactive oxygen species (hsa05208) significantly impact the occurrence and progression of liver cirrhosis. In order to investigate the mechanism of WLP in treating liver cirrhosis at a systemic level, we developed a comprehensive signaling pathway using these four key molecular pathways (Fig. 8).

To determine the position of WLP targets within these pathways, the first three columns were designated as upstream positions, while the remaining columns represented downstream positions. Among these pathways, the non-alcoholic fatty liver pathway (hsa04932) emerged as one of the top pathways influenced by KGEC in WLP for treating liver cirrhosis. KGEC regulates nine upstream targets, including Rac1, Cdc42, and TNFα, as well as 12 downstream targets such as JNK1, AP1, and IL-1. Upstream targets TNFα and JNK1/2 activate c-Jun and AP1 proteins, resulting in the production of downstream inflammatory factors like IL-6 and IL-1, which are closely associated with cell death in the steatosis liver observed in non-alcoholic fatty liver disease. Cell death can further progress non-alcoholic fatty liver disease into liver cirrhosis [49]. KGEC also regulates targets involved in chemical carcinogenesis-active oxygen (hsa05208), appearing both upstream and downstream of the pathway. These targets include EGFR, PTP18, and Src. Furthermore, Fig. 8 demonstrates that KGEC can influence the activation of p38 and AP1 proteins downstream of the non-alcoholic fatty liver pathway, impacting the treatment of liver cirrhosis. Therefore, KGEC in WLP plays a pivotal role in the treatment of liver cirrhosis by modulating the chemical carcinogenesis-reactive oxygen species-non-alcoholic fatty liver pathway, which synergistically affects tumor proliferation, angiogenesis, metastasis, and the development of liver disease.

The cAMP signaling pathway (hsa04024) and thermogenesis (hsa04714) are also significant pathways targeted by WLP for the treatment of liver cirrhosis. Many of the targets influenced by WLP are located downstream of these pathways. For instance, RAS, RSK, and PRAS40 in KGEC are downstream targets of thermogenesis (hsa04714). WLP may modulate upstream targets CB1R and GRB2, leading to the activation of downstream targets PKA and RAS. This, in turn, affects a range of signal transduction-related proteins like CREB, LSD1, PPARγ, mTOR, and PRAS40, all of which are associated with liver cirrhosis. In the cAMP signaling pathway (hsa04024), JNK, PDE, and CREB are downstream targets. KGEC can activate cAMP through upstream target ANP, influencing various genes related to cell proliferation, survival, and metabolism such as PI3K and Akt [50]. This modulation impacts hepatocyte insulin resistance and the occurrence of non-alcoholic fatty liver disease. Consequently, KGEC in WLP can synergistically contribute to the development of cirrhosis by regulating the ANP-Akt key cascade, playing a significant role in the treatment of cirrhosis.

### Verification the KGEC targets in vivo

**Fig. 9:**
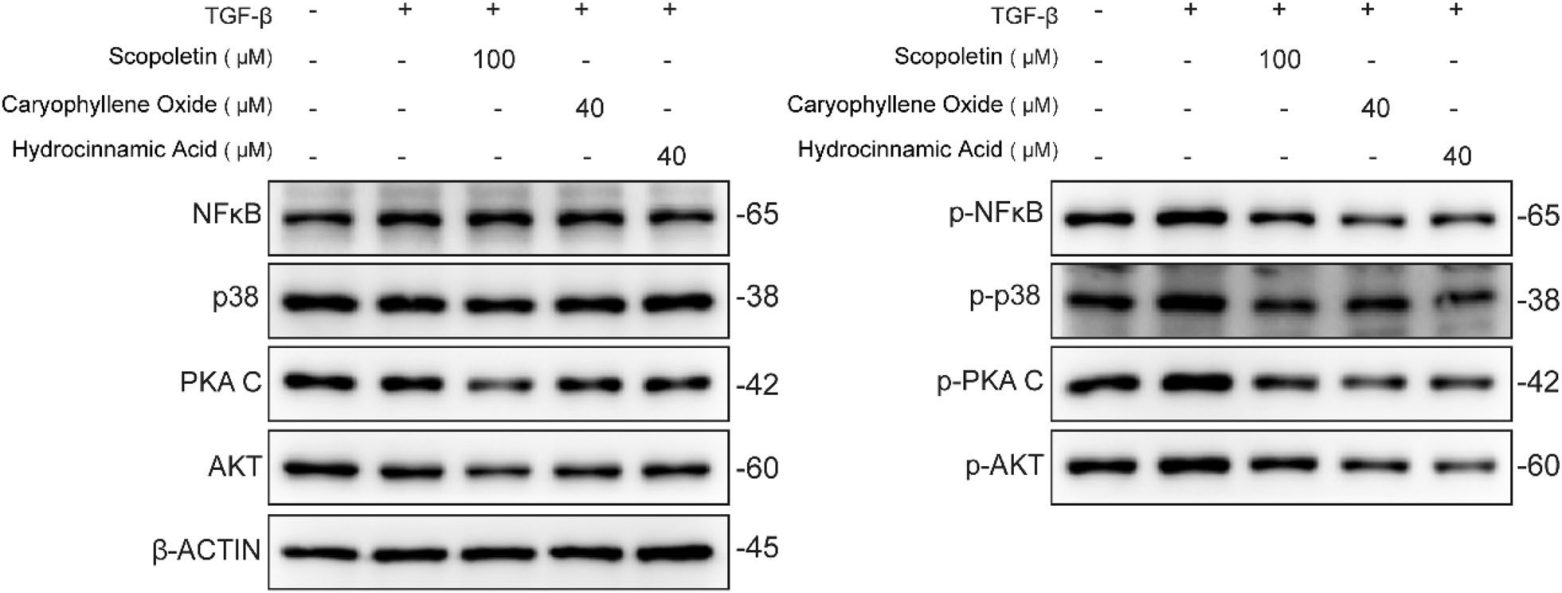
Verification the KGEC targets in vivo The protein expression levels of NF-κB, p-NF-κB, p38, p-p38, PKA C, p-PKA C, AKT and p-AKT were determined by western blot. β-ACTIN is shown as a loading control. (Full-length blots/gels are presented in Additional file 4: Figure S1)

Furthermore, to validate the comprehensive pathway results, we assessed the impact of the three components scopoletin, caryophyllene oxide and hydroxyzinamic acid on the NF-κB, AMPK/p38, cAMP, and PI3K/AKT pathways through western blot. The results showed that, as compared to the control group, all four pathways were activated in TGF-β1-induced LX2 cells. However, treatment with scopoletin (100 uM), caryophyllene oxide (40 uM), and hydroxyzinamic acid (40 uM) led to the suppression of the expression of p-NF-κB, p-p38, p-PKA C, and p-AKT (Fig. 9). This suggests that KGEC exerts its anti-liver fibrosis effects, at least in part, by targeting these four pathways. This further validates the reliability of our new model and the accuracy of KGEC.

## Discussion

The role of TCM formulas in treating complex diseases is increasingly recognized, as it possesses the characteristics of multiple components and targets. These component targets form a complicated network, and detecting the FRS and KGEC in this network are primary for understanding the molecular mechanism of TCM treatment.

To improve the clinical effectiveness of classical prescriptions, we utilized the computational pharmacology methodology to gain the most important associations between component targets and pathogenetic genes. An optimization calculation method was designed to obtain the important components of the TCM prescription WLP. At present, many researches are based on network topology such as degree, betweeness and closeness to obtain important components. However, these methods do not consider the biological significance of nodes and may miss some components with important biological functions. Therefore, this study uses multi-objective optimization algorithm and genetic knapsack algorithm to solve this problem. Multi-objective optimization algorithm is a mathematical method to find the solution with the minimum deviation from the target value under given constraints. In this study, we not only consider the topological structure of the network, but also the target of components and disease genes. Using this method, we obtained the FRS. Then the genetic knapsack algorithm is used to further obtain the KGEC. Genetic knapsack algorithm can search for the entire solution space and find the global optimal solution without getting stuck in local optimal solution, so it can comprehensively select the chemical components with therapeutic effects to achieve the optimisation of TCM formulas. Finally, the potential mechanism of WLP for the treatment of liver cirrhosis was inferred by KEGG and GO enrichment analysis. Specifically, the advantages of our model are as follows.

We adopted a new strategy to solve the problems of component targets network redundancy and the neglect of the cascade transmission between component targets and pathogenetic genes. First, we considered that PPI networks connect component targets and pathogenetic genes, so a component-target-pathogenetic gene network was constructed based on PPI networks. Then, we designed the optimization conditions of the multi-objective optimization method and used the degree attribute of the C-T network node to obtain some relatively important relationships between component targets, and thus constructed and verified the FRS. In the CAP pathways, the coverage rate of effective proteins enrichment pathways defined from the FRS was the highest, and they could not be replaced by disease-specific targets or component-specific targets. It confirms that the effective proteins we defined from the FRS act as a key role in the pathogenesis of liver cirrhosis.

The genetic knapsack algorithm was used to deduce KGEC from the effective proteins within the FRS and conduct functional analysis and verification. The total number of KGEC target enrichment pathways was 177, accounting for 83.33% of CAP pathways. This result confirms that the functional coverage of the extracted KGEC is improved after optimization by the genetic knapsack algorithm, and the molecules with invalid or weak effects are removed. This also indicates the reliability of our FRS and KGEC selection strategy.

KGEC has a total of 29 TCM active components. These active components affect the progression of cirrhosis in various ways, thereby alleviating liver function damage. One research suggested that GLY (WLP209) can reduce liver damage in rats with liver fibrosis and cirrhosis by affecting the levels of vasoactive substances ET-1, CGRP and ANP [51]. Another study found that HCI (WLP163) has antioxidant function and can significantly reduce oxidative stress in the liver [52]. Protocatechuic acid (WLP193) [53] significantly alleviates fatty acid metabolism disorder in high-fat diet-induced nonalcoholic fatty liver disease. Scopoletol (WLP219) [54] significantly down-regulates TLR4 signaling genes in WAT and liver, such as MyD88, TRIF, NF-κB, TNF-α, and IL-6, which play an important role in improving alcohol-induced lipid dysregulation and inflammation. In summary, these key components are important in the treatment of cirrhosis by WLP and work in a variety of different pathways. To speculate on potential therapeutic mechanisms, the targets of the 29 components were analyzed using GO and KEGG. In GO analysis, WLP regulatory targets are involved in mitochondrial electron transfer, respiratory chain complex and dehydrogenase complex, and the regulatory molecular functions include oxidation–reduction reaction-related enzyme activity, etc. In KEGG analysis, four relatively important pathways were identified. Among them, non-alcoholic fatty liver disease (hsa04932) can further aggravate non-alcoholic fatty liver disease into liver cirrhosis [55]. Thermogenesis (hsa04714) can occur in up to 18% of patients with cirrhosis, whose increased resting energy consumption is associated with deteriorating hepatic circulation [56]. cAMP signaling pathway (hsa04024) can be inhibited during liver injury. cAMP has the capacity to regulate multiple cell functions, including lipid metabolism, inflammation, cell differentiation and injury [57]. In chemical carcinogenesis-reactive oxygen species (hsa05208), which enhances oxidative stress in hepatic parenchymal cells, explains the close relationship between hepatic fibrosis and the occurrence of liver cancer [58]. We found that key component groups in WLP synergistically affected the occurrence of cirrhosis by regulating the chemical carcinogenesis-reactive oxygen species-non-alcoholic fatty liver pathway and regulating the critical cascade of ANP-Akt.

## Conclusion

A computational pharmacology model-based bioinformatics algorithm was established to extract the KGEC of WLP and predict the action mechanism of WLP in treating liver cirrhosis. In comparison to other works, this paper constructs FRS based on the multi-objective optimization method and uses the genetic knapsack algorithm to screen KGEC from the FRS. Based on fundamental pharmacological data, this research is a work of computational data analysis, aiming to provide a practicable strategy for elucidating the molecular mechanism of TCM in treating complex diseases and avoiding large-scale experimental verification.

However, this study also has certain limitations. There is only one KGEC selected for reliability testing, and no additional component groups are included. Moreover, the predicted mechanism of WLP in treating liver cirrhosis has not been further verified in animal experiments.

### Supplementary Information

**Additional file 1: **The primers for qPCR experiments.

**Additional file 2: **The pathogenic genes of liver cirrhosis.

**Additional file 3: **The active components of WLP.

**Additional file 4:** **Figure. S1.** The full-length blots of NF-κB, p-NF-κB, p38, p-p38, PKA C, p-PKA C, AKT, p-AKT and β-ACTIN

## Data Availability

All data related to this study are included in the article/supplementary material and further inquiries can be directed to the corresponding author.
