# Supplementary material for: A novel network pharmacology strategy to decode mechanism of Wuling Powder in treating liver cirrhosis
Source: Chin Med. 2024 Mar 1;19:36. doi: 10.1186/s13020-024-00896-z (PMC10905787; doi:10.1186/s13020-024-00896-z)
Supplement: Supplementary file 4 — Additional file 4: Figure. S1. The full-length blots of NF-κB, p-NF-κB, p38, p-p38, PKA C, p-PKA C, AKT, p-AKT and β-ACTIN [file 13020_2024_896_MOESM4_ESM.docx]

**Supplementary Materials for**

A Novel Network Pharmacology Strategy to Decode Mechanism of Wuling Powder in Treating Liver Cirrhosis

Qinwen Liu^1,2#^, Xiaowei Li^1,2#^, Yi Li^1,2^, Qian Luo^1,2^, Qiling Fan^1,2^, Aiping Lu^5,6*^, Daogang Guan^1,2*^, Jiahui Li^1,3,4*^


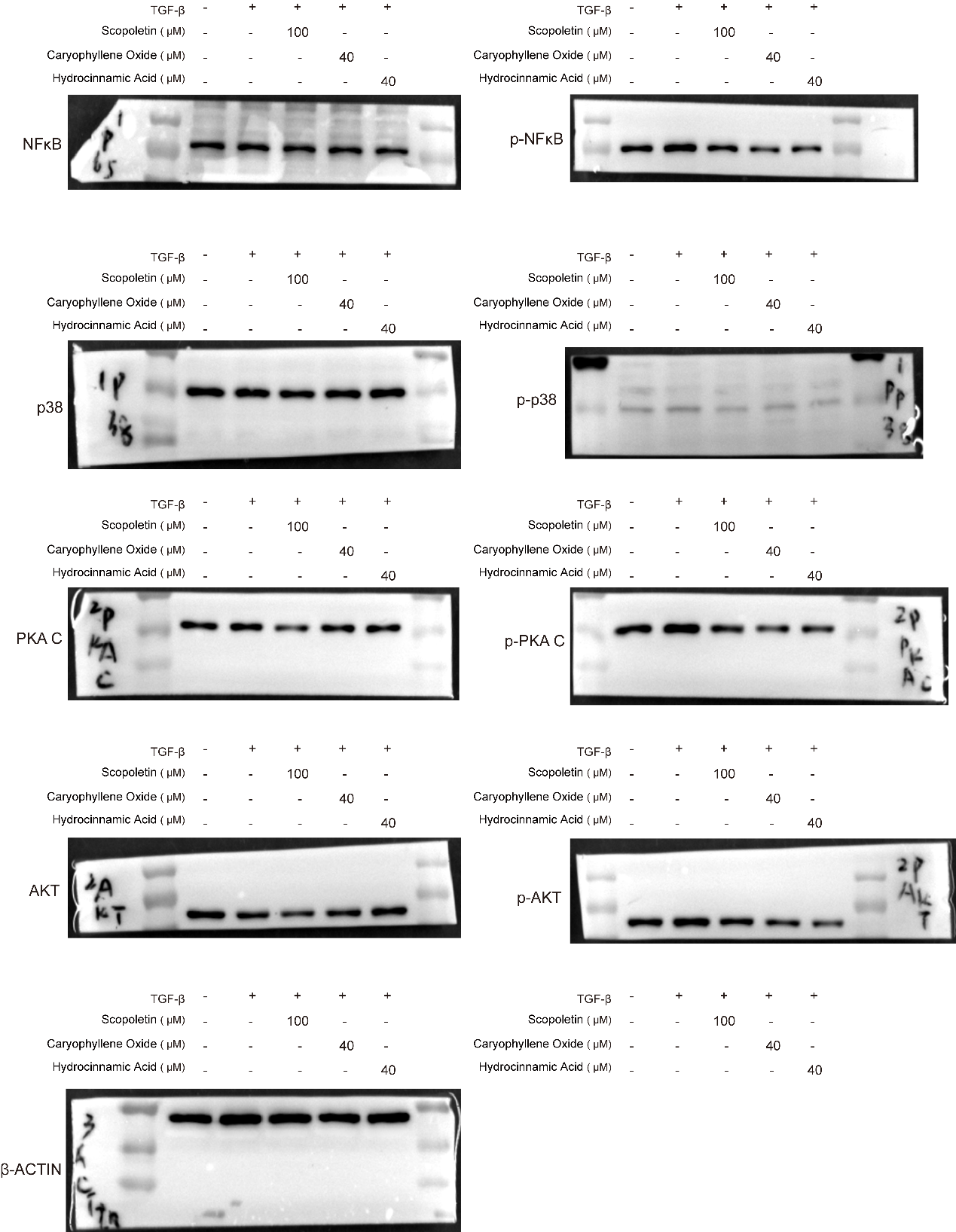
**Figure. S1.** The full-length blots of NF-κB, p-NF-κB, p38, p-p38, PKA C, p-PKA C, AKT, p-AKT and β-ACTIN.
